# Primary care provider preferences for working with a collaborative support team

**DOI:** 10.1186/1748-5908-2-16

**Published:** 2007-05-30

**Authors:** Steven K Dobscha, Ruth Q Leibowitz, Jennifer A Flores, Melanie Doak, Martha S Gerrity

**Affiliations:** 1 Columbia Center for the Study of Chronic, Comorbid Mental and Physical Disorders, Portland VA Medical Center, Portland, Oregon, USA; 2 Department of Psychiatry, Oregon Health & Science University, Portland, Oregon, USA; 3 Primary Care Division, Portland VA Medical Center, Portland, Oregon, USA; 4 Department of Medicine, Oregon Health & Science University, Portland, Oregon, USA; 5 Division of Hospital and Specialty Medicine, Portland VA Medical Center, Portland, Oregon, USA

## Abstract

**Background:**

Clinical interventions based on collaborative models require effective communication between primary care providers (PCPs) and collaborative support teams. Despite growing interest in collaborative care, we have identified no published studies describing how PCPs prefer to communicate and interact with collaborative support teams. This manuscript examines the communication and interaction preferences of PCPs participating in an ongoing randomized clinical trial of a collaborative intervention for chronic pain and depression.

**Methods:**

The trial is being conducted in five primary care clinics of a Veterans Affairs Medical Center. Twenty-one PCPs randomized to the study intervention completed a survey regarding preferences for interacting with the collaborative support team.

**Results:**

A majority of PCPs identified email (95%) and telephone calls (68%) as preferred modes for communicating with members of the support team. In contrast, only 29% identified in-person communications as preferred. Most PCPs preferred that the care manager and physician pain specialist assess patients (76%) and make initial treatment changes (71%) without first conferring with the PCP. One-half wanted to be designated cosigners of all support team notes in the electronic medical record, one-half wanted to receive brief and focused information rather than in-depth information about their patients, and one-half wanted their practice nurses automatically included in communications. Panel size was strongly associated (p < 0.001) with preference for brief, to-the-point discussions about patients.

**Conclusion:**

The substantial variation in PCP communication preferences suggests the need for knowledge of these preferences when designing and implementing collaborative interventions. Additional research is needed to understand relationships between clinician and practice characteristics and interaction preferences.

## Background

Multifaceted clinical interventions based on the chronic care model [1], also known as collaborative interventions, have been shown to improve outcomes of a variety of illnesses treated in primary care, including depression, diabetes, asthma, and hypertension [1-10]. In the chronic care model, productive interactions between patients and primary care providers (PCPs) are at the core of successful clinical outcomes. Collaborative interventions for chronic illnesses are evidence-based, and typically involve patient and provider activation and education, monitoring of clinical outcomes over time by a collaborative support team, provision of recommendations, feedback and supplemental education to clinicians by the support team, and modifications in information systems [1]. Collaborative support teams typically consist of a care manager and supervising chronic disease expert physician. Effective communication among the PCP, members of the support team, other members of the care team (e.g., clinic staff nurses), and patients is critical to the success of the collaborative approach.

While several published studies have documented selected clinician attitudes and satisfaction regarding disease management and collaborative care approaches, they do not specifically report on how PCPs prefer to communicate or interact with members of collaborative support teams [11-16]. This manuscript describes the communication and interaction preferences of PCPs participating in a randomized trial of a collaborative intervention for chronic pain and depression, and examines associations between PCP characteristics and their preferences.

## Methods

The study of the effectiveness of a collaborative approach to pain (SEACAP) is an ongoing randomized clinical trial being conducted in five primary care clinics of a Veterans Affairs (VA) medical center [17]. One of the clinics (21,000 patients and 34 staff PCPs) is located at the main medical center site in an urban location. Two clinics (8,000 patients and 13 staff PCPs; 4,400 patients and 6 staff PCPs) are located in urban areas 12 and 50 miles from the medical center, respectively. The other two clinics (1,300 patients and 2 staff PCPs; 4,200 patients and 5 staff PCPs) are located in rural areas 95 and 175 miles from the medical center, respectively. Some PCPs have teaching, research, or administrative responsibilities, which creates substantial variation in the amount of time spent in clinic and in panel sizes.

SEACAP is testing whether the collaborative intervention, Assistance with Pain Treatment (APT), improves pain and depression-related patient outcomes compared to treatment as usual (TAU). APT is based on previously studied interventions, and includes clinician education, patient education and activation, symptom assessment, and outcomes monitoring, with ongoing feedback and recommendations to PCPs provided by a collaborative support team. The collaborative support team consists of a 1.0 FTEE psychologist care manager and 0.2 FTEE primary care physician with supplemental training in chronic pain.

Forty-two (84%) of fifty eligible full- and part-time staff PCPs agreed to participate in SEACAP. Twenty (48%) of these PCPs had previously participated in a randomized trial of a collaborative approach to depression. Participating PCPs were randomized to receive the APT intervention versus TAU. There were no significant differences in demographic and practice characteristics when comparing intervention to TAU clinicians. Intervention clinicians (n = 21) then completed a survey regarding their preferences for communicating and interacting with the collaborative support team (Appendix A).

Response options for preferred modes of communication were not exclusive. Information on PCP panel size was obtained from the local electronic record (VISTA) for the month (December 2005) prior to initiating the SEACAP trial. Fisher's exact test was used to test for associations between PCP characteristics and preference survey item responses (all binary), and for correlations within preference survey responses.

## Results

Table 1 describes characteristics of the 21 intervention PCPs, as well as their preferences for interaction. Two-thirds of the PCPs were physicians, and two-thirds were female. As expected, there was considerable variation in panel size.

**Table 1:** Primary Care Provider Characteristics and Preferences (n = 21)

**Clinician Characteristic**	**Value**
Physicians, n (%)	14 (67%)
Nurse Practitioners or Physician Assistants, n (%)	7 (33%)
Female, n (%)	14 (67%)
Mean years since training, yr (sd)	18 (9.5)
Panel size	
Mean number of patients in panel, n (%)	700 (385)
Median panel size	811
Practicing in rural clinic, n (%)	4 (19%)
**Preferences**	
Mode of Communication^1^, n (%)	
E-mail	20 (95%)
Telephone or pager	14 (67%)
In-person discussion	6 (29%)
Prefers in-depth discussions of patient when time permits, n (%)	11 (52%)
Prefers intervention team assess patient without contacting PCP first, n (%)	16 (76%)
Prefers to co-sign all intervention team notes in the electronic record, n (%)	11 (52%)
Prefers clinic nurse automatically included in intervention communications, n (%)	10 (48%)
Prefers intervention team write orders without discussing changes first, n (%)	15 (71%)
Prefers intervention team write new initial medication orders^2^, n (%)	15 (83%)

^1^PCPs were asked to identify all preferred modes of communication.

^2^Three PCPs did not respond or had ambiguous responses to this item

Most PCPs identified email (95%) and telephone calls (68%) as preferred modes for communicating with members of the support team, while only 29% identified in-person communications as preferred. Most PCPs preferred that the support team assess patients (76%) and make initial treatment changes (71%) without first conferring with the PCP. One-half of the PCPs wanted to be designated cosigners of all support team notes in the electronic medical record, one-half wanted to receive brief and focused information rather than in-depth information about their patients, and one-half wanted their practice nurses automatically included in communications.

There was a strong inverse association between panel size and preference for in-depth discussion of cases. None of the PCPs with panel sizes above the median (≥ 811) preferred in-depth discussions, while 10/12 (83%) PCPs with panel sizes below the median preferred more in-depth discussions when time permits (Fisher's exact p < 0.001). We also found a marginally significant association between gender and preference for discussing treatment before initiating changes: 6/14 (43%) of female PCPs preferred to discuss treatment first, while none of seven male PCPs preferred to discuss treatment first (Fisher's exact p = 0.061). There were no significant differences in preferences of PCPs when comparing physician to nurse practitioner and physician assistant responses, or when comparing rural to urban responses.

Two significant correlations were found within the survey item responses. Preference for cosigning all intervention team notes was positively associated with preference for telephone or pager communication (Fisher's exact p = 0.024), and with preference for being contacted before intervention team assessment of patients (Fisher's exact p = 0.035).

## Discussion

Our findings show that there is considerable variation in the preferences of PCPs for interacting with collaborative support teams, especially with regard to brief versus in-depth discussion about patients, receiving electronic alerts and cosigning notes, and involving clinic nurses in care team communications. Importantly, while most PCPs prefer that the support team proceed to assess patients and initiate treatment without prior discussion, a minority prefer to be more actively involved in developing and initiating treatment changes.

We found several correlates of preferences for more or less direct involvement in collaborative care. While it is not surprising that clinicians with larger panel sizes are less likely to prefer in-depth discussion about their patients, it was surprising to find that none of the clinicians with larger panels prefer in-depth discussions, even though we specified "when time permits" in our question. This finding likely reflects the substantial burden on full-time primary care clinicians, and the challenges faced by PCPs in responding to multiple sources of information.

We also found that female PCPs prefer to discuss initial treatment recommendations more frequently than their male colleagues. This finding cannot be explained by panel size, since female PCPs were not more likely to have smaller panel sizes. Previous studies have shown that male and female physicians have differing communication and interaction styles [18,19]. While these studies examined physician-patient interactions, it is reasonable to expect that gender-related communication and decision-making styles would influence preferences for interacting with collaborative support teams. Further exploration of the relationships among gender, other clinician and practice characteristics, and preferences for interaction, using a larger clinician sample, is indicated.

The variability we found in interaction preferences suggests that a "one size fits all" approach to collaborative care communication procedures may not be as satisfying for PCP participants as an individualized approach. In addition, responsiveness of PCPs to collaborative support recommendations might be enhanced if individual communication plans were used for each of the participating PCPs. Because of these possibilities, the results of the PCP preferences survey have been incorporated into routine SEACAP support team procedures. Preference information is entered into the support team's database. When a particular patient record is accessed, the preferences of his or her PCP are shown on the same screen, and this information is used to guide interactions with the PCP.

However, there may be potential downsides to using an individualized approach to collaborative support team communications with clinicians. If not designed and delivered carefully, an individualized communication plan could increase the likelihood of deviation from a standardized, evidence-based treatment algorithm. Loss of efficiency might arise from a support team having to develop multiple communication pathways. Clearly, more study is needed of the impact of individualized collaborative care communication approaches on clinician satisfaction and intervention effectiveness.

Finally, guideline-level care is challenging to implement in clinical settings, and lack of clinician motivation and buy-in can be important barriers [12,15,20]. Systematically inquiring about clinician preferences during development and implementation of collaborative care programs has the potential to enhance buy-in. In addition, information learned during the survey process may allow for clarification of misconceptions or idealizations about the intervention. Data from the Physician-System Alignment study showed that active physician participation in the implementation phase of care management was positively associated with subsequent attitudes and participation in care management activities [12,21]. However, this study also indicated that active participation of clinicians in the development phase was negatively associated with subsequent attitudes and participation.

The main limitation of the current study is our small sample size, which limited subgroup analyses and precluded factor analysis of our survey. Indeed, our preliminary analysis of item correlations suggests that there may be key factors or clinician interaction styles that might be identifiable if a larger sample were available. Another key limitation is that generalizability of our findings may be limited due to the particular group of clinicians studied.

## Conclusion

The results of this study show that there is considerable variation in PCP preferences for interacting with a collaborative support team. Although most PCPs indicate a desire for support team members to proceed with assessment and treatment without their preliminary input, a minority of PCPs want more direct involvement in treatment. Finally, the relationships between clinician and practice characteristics, and the effects of incorporating clinician preferences into intervention design and implementation, need to be studied further.

## Competing interests

The author(s) declare that they have no competing interests.

## Authors' contributions

SD was the primary author, contributed to study design, and confirmed the results. RL developed the preference questionnaire, contributed to study design, and revised the manuscript critically for important content. JF performed the data analyses and revised the manuscript critically for important content. MD developed the preference questionnaire, and revised the manuscript critically for important content. MG contributed to study design and revised the manuscript critically for important content. All authors read and approved the final manuscript.

## Appendix A

### Survey of PCP preferences for the assistance with pain treatment intervention

MODE OF COMMUNICATION:

Please check all preferred modes of communication and fill in the relevant information.

□ Telephone: ________________________

□ Outlook e-mail

□ Vista e-mail/GUImail

□ Pager: __________________

□ In-person discussion

□ Other: _____________________________

**COMMUNICATION STYLE **(please check all that apply)**:**

When communicating with me about patients:

□ I prefer brief, to-the-point communication that does not go into great detail about the

patient but instead focuses on a particular problem or issue and potential approach.

□ *When time permits *I prefer to have in-depth discussions about patients that include

details about associations between psychosocial and medical issues.

□ I prefer that you include my clinical nurse manager in communications regarding our

patients. Name of nurse manager: ___________________________________

□ Other: _______________________________________________________________

PSYCHOSOCIAL ASSESSMENT:

□ Please contact me prior to beginning your assessments with my patients–I may have

particular concerns or questions related to patients I might like for you to address in your assessment.

□ I would prefer for you to proceed with the assessment process for my patients without my initial feedback, assuming the clinical situation appears fairly straightforward.

CPRS (Electronic Medical Record) NOTES:

□ Please add me as a co-signer on all your notes related to my chronic pain patients

□ Add me as a co-signer only on notes that indicate an important change in the patient's

behavior or health status or suggested changes in treatment plan.

□ Please include clinical nurse manager as co-signer on notes regarding this patient.

Name of nurse: _____________________________________________

ORDERS/MEDICATION CHANGES (NON-OPIOID):

□ I prefer that you discuss any changes with me prior to making them.

□ I prefer to order meds myself.

□ I prefer for you to order meds initially; I will then take over prescribing.

□ I prefer for you to order meds under my name as an unsigned order, and I will sign.

□ Other preference not listed above:
